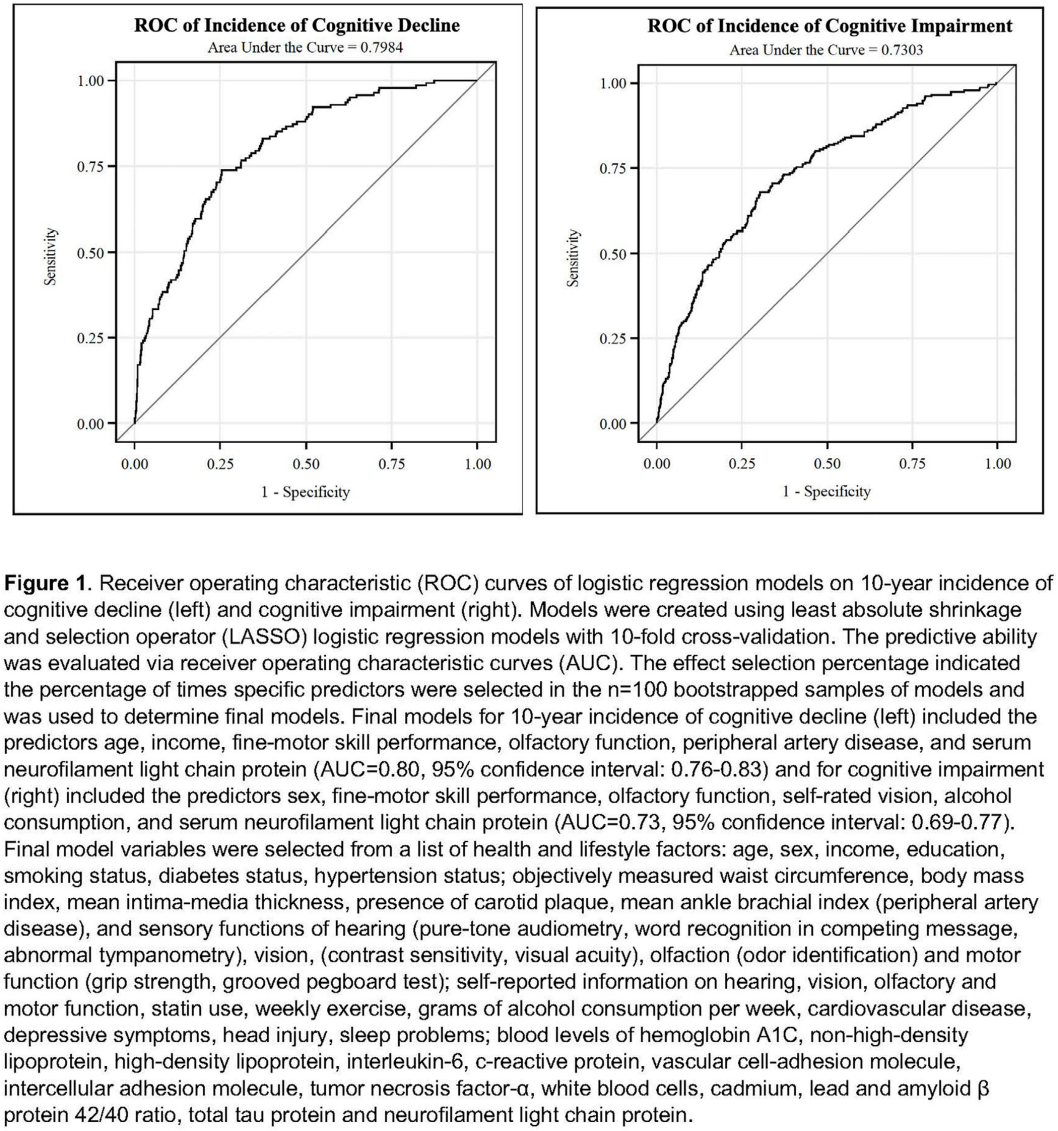# Midlife Sensory and Motor Measures Among Best Predictors in Parsimonious Models of Long‐Term Cognitive Decline and Onset of Cognitive Impairment in Middle‐Aged Adults

**DOI:** 10.1002/alz.086737

**Published:** 2025-01-03

**Authors:** Natascha Merten, A Alex Pinto, Adam J Paulsen, Richard J Chappell, Yanjun Chen, Corinne D. Engelman, Laura M Hancock, Sterling C. Johnson, Carla R Schubert

**Affiliations:** ^1^ University of Wisconsin‐Madison, Madison, WI USA; ^2^ Cleveland Clinic, Cleveland, OH USA; ^3^ School of Medicine and Public Health, University of Wisconsin‐Madison, Madison, WI USA

## Abstract

**Background:**

Over the past decades, many risk factors for dementia have been identified including sensory and motor functions. Established risk scores to predict onset of cognitive impairment and/or dementia (e.g., the CAIDE and Framingham Risk Score) often focused on cardiovascular risk factors and have not been updated in more recent generations. Risk scores for asymptomatic middle‐aged people, which are based on practical test batteries will be useful for targeted population screenings. The aim of this study was to construct a parsimonious risk prediction model of 10‐year cognitive decline and impairment using factors measured in midlife in a current cohort.

**Method:**

This longitudinal study is based on N = 1529 (54% women, mean age 49 years) Beaver Dam Offspring Study participants with data from baseline, 5‐year and 10‐year follow‐up. We assessed objectively measured and self‐reported hearing, vision, olfactory, and motor function, lifestyle, cardiovascular and general health factors, and blood‐based markers of inflammation, neurodegeneration, and amyloid. We determined 10‐year cognitive decline (trail‐making test B time;10% most decline) and cognitive impairment (neurocognitive case review). We constructed least absolute shrinkage and selection operator (LASSO) logistic regression models with 10‐fold cross‐validation and evaluated predictive ability via receiver operating characteristic curves (AUCs). The effect selection percentage indicated the percentage of times specific predictors were selected in the n = 100 bootstrapped samples of models and was used to establish final models.

**Results:**

There were N = 121 cognitive decline and N = 217 cognitive impairment cases. The six top predictors for cognitive decline (age, income, fine‐motor skill performance, olfactory function, peripheral artery disease, serum neurofilament light chain protein (NfL)) and for cognitive impairment (sex, fine‐motor skill performance, olfactory function, self‐rated vision, alcohol consumption, NfL) yielded models with an AUC of 0.80(95% confidence interval:[0.76‐0.83]) and 0.73[0.69‐0.77], respectively.

**Conclusion:**

In middle‐aged adults, measures of sensory and motor function and NfL were among the best predictors of 10‐year onset of cognitive decline and impairment and only 6 factors were needed to achieve acceptable to excellent AUCs. Cross‐validation in another cohort is needed to verify these factors as reliable and valid predictors to identify those at high risk for neurodegeneration and cognitive decline who could benefit from early prevention and intervention strategies.